# Patient Satisfaction and Quality of Recovery With Ambulatory Serratus Plane Catheter After Mastectomy: A Service Evaluation

**DOI:** 10.7759/cureus.52588

**Published:** 2024-01-19

**Authors:** Goran A Ahmed, Franklin Wou, Rishabha D Sharma, Madan Narayanan

**Affiliations:** 1 Breast Surgery, Frimley Health NHS Foundation Trust, Surrey, GBR; 2 Anesthesiology and Critical Care, Guy's and St Thomas' NHS Foundation Trust, London, GBR; 3 Anesthesiology and Critical Care, Frimley Health NHS Foundation Trust, Surrey, GBR; 4 Breast Surgery, University Hospitals Plymouth NHS Trust, Plymouth, GBR

**Keywords:** mastectomy, ambulatory infusion pump, regional block, nerve block, mibr, day-case mastectomy, serratus block

## Abstract

Background

Acute moderate to severe pain after mastectomy is common and impedes patient recovery. Ambulatory serratus plane catheter with infusion pump (ASPIP) is a novel method to provide continuous delivery of local anaesthetic agents in the immediate postoperative period for extended analgesia, early mobility, and return to function after mastectomy. The aim of this project was to evaluate the introduction of ASPIP service and its effect on postoperative pain, opioid use, hospital stay, and recovery.

Methods

This was a service evaluation project. Eligible mastectomy patients were included over six months. All patients provided consent for intraoperative catheter insertion and ASPIP use. The numerical rating scale (NRS) and the Quality of Recovery-15 (QoR-15) tool were used to assess postoperative pain and the quality of recovery, respectively. Overall satisfaction, sleep quality, and recommendations for the catheter were collected. Day-case rates of mastectomy with or without reconstruction were also measured. Data were presented using descriptive statistics. Mean (SD) and median (quartiles) were used for the continuous variables with percentages to report rates. Approval of the institution's Audit & Quality Improvement Department was obtained.

Results

Thirty-two consecutive mastectomy patients were included over six months. The mean age was 60 years and the mean BMI was 25.8. Mean pain NRS (10 maximum) at rest and on movement were 1.88 vs. 2.56, 2.03 vs. 2.84, and 1.85 vs. 2.3 out of 10 on postoperative day (POD) one, two, and three, respectively. Six patients required opioids on POD one, four patients on POD two, and none on the third day. Sleep disturbance was observed in three, five, and three patients in the first three days, respectively. The mean overall satisfaction was 9.25/10 (SD: 1.18). All patients recommended ASPIP to other patients. QoR-15 scores reported as median (quartiles) were 143 (136, 147) preoperatively and 135 (126.5, 143) postoperatively, with a median difference of -3 (95% CI: -6 to 0). The day-case rate for mastectomy +/- reconstruction was 66% and 39%, respectively. There were no major complications from the catheter with minor issues in four patients.

Conclusion

The ASPIP is an effective and safe method of managing postoperative pain after mastectomy with positive patient experience and reduced opioid requirement. As part of an enhanced recovery pathway, it can also increase mastectomy day-case rates, including immediate implant reconstruction.

## Introduction

Acute pain after mastectomy remains a common problem leading to slow recovery, delayed ambulation and return to normal activities, and an increased length of hospital stay and re-attendance [1]. Evidence suggests that the severity of acute postoperative pain is strongly associated with time to recovery and opioid cessation [2]. Poorly controlled acute pain directly correlates with decreased quality of recovery in the postoperative period and in addition, increases the risk for the development of chronic pain syndromes [3,4]. Significant acute pain after mastectomy can progress to chronic pain in 25-60% of patients [5].

In recent years, there has been a move towards regional nerve blocks to reduce opiate use and postoperative nausea and vomiting and facilitate early mobility and discharge after breast surgery. A variety of chest wall blocks have been used and investigated, including paravertebral, interpectoral plane block (formerly Pecs 1), and pectoserratus plane block (formerly Pecs 2), which are all effective in managing postoperative pain [6,7]. These are, however, not without risks and serious complications, including pneumothorax and nerve damage, with thoracic paravertebral and intercostal blocks carrying the highest risk [8,9].

Serratus anterior plane block (SAPB) has been shown to be a safe and effective method to manage perioperative pain in breast surgery [10,11]. Studies have shown SAPB to cause chest wall analgesia lasting for 12 hours following breast surgery [12,13]. First described by Blanco et al. in 2013, this block targets the lateral cutaneous branches of the intercostal nerves, providing analgesia to the anterolateral chest wall [14]. It involves local anaesthetic infiltration in the plane between superficial serratus fascia, latissimus dorsi muscle postero-laterally, and pectoralis minor muscle anteriorly. The serratus anterior muscle is easily identified during mastectomy in the anterolateral chest wall. This makes infiltration or placement of a catheter by the operating surgeon, under direct vision, an easy task.

Insertion of an indwelling catheter into the serratus plane to deliver continuous local anaesthetic agents in breast surgery is a novel method with very few prior studies in the context of mastectomy [15,16]. Catheters attached to ambulatory pumps provide continuous delivery of local anaesthetics, longer lasting analgesia, and improve patient mobility and their ability to go home on the same day.

In this quality improvement initiative, we aimed to evaluate the overall satisfaction, pain scores, and quality of recovery (QoR) using the ambulatory serratus plane catheter with infusion pump (ASPIP) after mastectomy.

We also aimed to measure the rate of day-case mastectomy in our cohort after the introduction of this service. The latter is especially important as the British Association of Day Surgery (BADS) encourages 75% of mastectomies in a unit to be done as day surgery [17]. In the latest Getting It Right First Time (GIRFT) report in the UK in 2021, our trust's day-case rate for simple mastectomy was 6% and no mastectomy and immediate breast reconstruction (MIBR) was done as day-case [18].

## Materials and methods

This was an audit and service evaluation of the use of ASPIP on patients who underwent mastectomy with or without breast reconstruction and axillary surgery between October 2021 and March 2022 at Frimley Park Hospital, UK.

All patients older than 18 years, who underwent mastectomy for breast cancer or risk reduction were consented and included in the service evaluation. Patients who had significant allergies to local anaesthetics or were unable to manage a local anaesthetic infusion pump at home either due to their cognitive function or language barrier or where the patient lived quite far away from the hospital with limited access to community teams were excluded from the service evaluation.

The main outcomes of interest were pain scores, opioid use, overall satisfaction, quality of sleep and quality of recovery (QoR), and the rates of day-case mastectomy with or without immediate reconstruction. Day-case surgery in the UK is defined as a patient being admitted for a procedure and discharged home on the same day. This is different from the '23-hour stay’ definition used in the United States [19].

**Figure 1:**
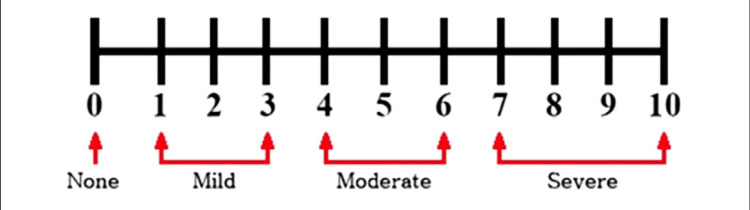
Numerical rating scale

Patients reported their pain scores at rest and on movement on days one, two, and three using the numerical rating scale (NRS), with 0 being no pain and 10 being the most severe pain (Figure 1). Patients reported the number of times they woke up during the night because of pain. Similarly, patients reported their overall satisfaction with the catheter based on pain management on a score between 0 and 10, with 0 being totally dissatisfied and 10 being totally satisfied.

Overall QoR was assessed using the validated Quality of Recovery-15 (QoR-15) questionnaire (see Appendix 1) [20]. The questionnaire is in two parts with a total of 15 questions, with patients scoring from 0 to 10 for each question to a total of 150. Patients completed the QoR questionnaire preoperatively as a baseline and on day two postoperatively for comparison.

The project was registered with the Quality & Audit Department of the trust (Frimley Park Hospital Clinical Excellence registration reference number: FH555). Additionally, it was approved by the Information Governance Department of the trust. All patients consented to the insertion of the catheter intraoperatively by the surgeon.

All operations were carried out under the supervision of three oncoplastic breast surgeons. Descriptive statistics using Microsoft Excel (Microsoft Corporation, Redmond, WA) was used to measure the outcomes and report the findings.

Anaesthetic and surgical technique

All patients received general anaesthesia according to the institution’s enhanced recovery programme for mastectomies. Anaesthesia was induced using propofol and a short-acting opioid and maintained either with total intravenous anaesthesia or sevoflurane at the discretion of the attending anaesthetist with a processed electroencephalogram to guide the depth of anaesthesia. In line with PROSPECT recommendations, all patients received a preoperative nerve block (paravertebral or pectoral nerve blocks) and multi-modal analgesia intraoperatively (paracetamol, non-steroid anti-inflammatory agents, and intravenous dexamethasone) [21]. The preoperative nerve block was to provide analgesia for a maximum of 12 hours with the aim of limiting opioid use and postoperative nausea and vomiting (PONV). This was thought not to confound the results as data were collected for 72 hours after surgery.

**Figure 2:**
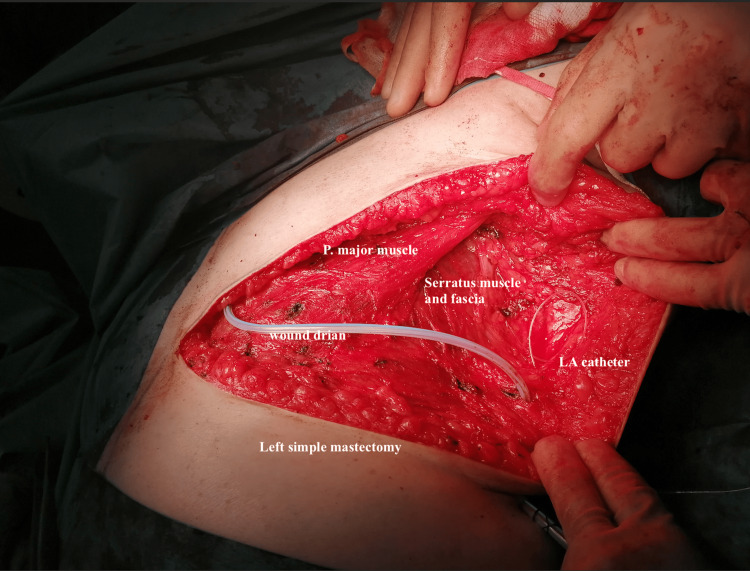
Intra-operative demonstration of catheter in a simple mastectomy wound

**Figure 3:**
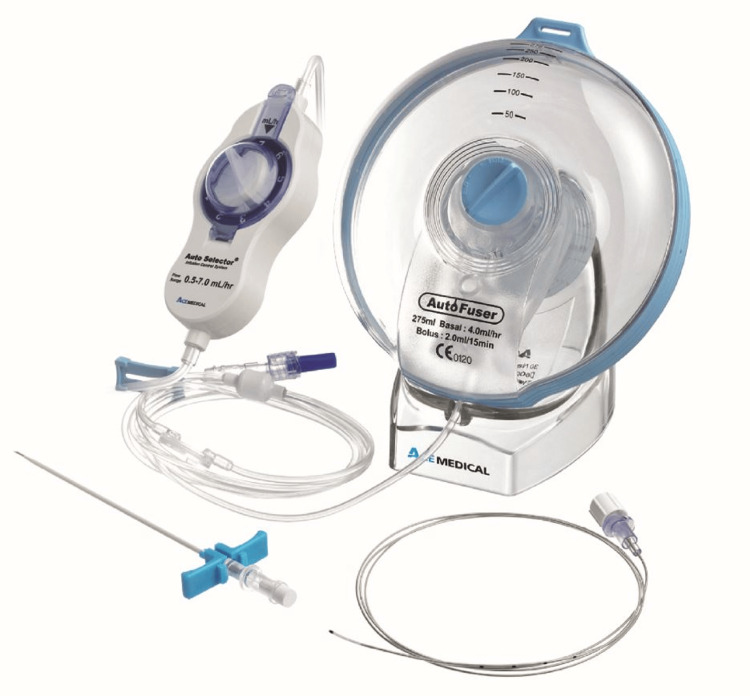
Ambulatory elastomeric pump used for continuous local anaesthetic infusion

Various incisions were used for mastectomy depending on whether it was simple, skin-sparing, or nipple-sparing mastectomy. Upon conclusion of the mastectomy and before wound closure, a 16G epidural Tuohy needle was inserted into the superficial serratus plane at the level of the 5th intercostal space and advanced cephalad towards the apex of the axilla (Figure 2). After hydro-dissection of the plane with 5 mL of normal saline, a 19G catheter was inserted 5 cm beyond the tip of the needle. The catheter was tunnelled through a separate exit point in the skin away from the incision and attached to an ambulatory infusion pump after the operation. Ten millilitres of 0.25% bupivacaine was injected down the catheter as an initial bolus dose. The elastomeric infusion pump ‘Auto Fuser’ (Ace Medical, Seoul, South Korea) was used in all cases. It includes a strap clip and carrying pouch for flexible ambulation (Figure 3). The pump was filled with 400 ml of 0.125% (1.25 mg/ml) levobupivacaine and commenced at an infusion rate of 6 ml/hr. This provided a continuous infusion of local anaesthetic over the next 66-67 hours. The dose per day was about 170 mg of levobupivacaine, which was well below the maximum recommended dose of 400 mg/day for levobupivacaine according to the British National Formulary (BNF).

Patients were discharged home with regular simple oral analgesics and a weak oral opioid (codeine) for rescue breakthrough pain.

Follow-up

The serratus catheter was removed from the patients on postoperative day (POD) two or three by the district nurses. Patients had a telephone follow-up by the anaesthetic regional fellow on days one, two, and three depending on when the catheter was due to be removed and the postoperative QoR-15 score was assessed on POD two. Additionally, patients were followed up by the breast care nurses for any problems related to the surgery, wounds and drain management.

All patients had a clinical face-to-face review in the outpatients after two to three weeks for a wound review and results of histology. Patients who had reconstruction were seen after one week for wound review.

We are currently conducting a follow-up survey of the patients who had the ambulatory serratus catheter and the effect of the catheter on the incidence of post-mastectomy pain syndrome.

## Results

The serratus plane catheter was used in 32 consecutive mastectomy (31 female and one male) patients between October 2021 and March 2022. Table 1 shows patient demographics, type of surgery, and outcomes. The patients in the table are in chronological order from October 2021 to March 2022. The mean age was 60 years and the mean BMI was 25.8.

**Table 1:** Patient characteristics, type of surgery, and postoperative outcomes BMI: body mass index; ASA: American Society of Anesthesiologists classification; LOS: length of stay; Mx: mastectomy; ANC: axillary node clearance; SNB: sentinel node biopsy; SS: skin-sparing; NS: nipple-sparing; DTI: direct to implant; prepec: prepectoral; recon: reconstruction; RTT: return to theatre.

Pt.	Age (years)	BMI (kg/m^2^)	ASA	Operation	LOS (days)	Complications	Readmission 30 days
1	68	27.8	2	Simple Mx + SNB	0	Nil	No
2	45	22.7	2	NS Mx + ANC + prepec reconstruction with expander	2	Nil	No
3	63	23.94	2	Simple Mx + SNB	0	Axillary seroma	No
4	67	34.5	2	Simple Mx + SNB	0	Nil	No
5	66 Male	26.2	2	Simple Mx + SNB	0	Seroma	No
6	71	24.6	2	Simple Mx + SNB	0	Nil	No
7	62	41.7	3	Simple Mx + SNB	0	Large seroma drained in clinic	No
8	35	21.6	2	NS Mx + SNB + implant recon prepec DTI	0	Nil	No
9	39	26.4	2	NS Mx + SNB + implant recon prepec DTI	0	Nil	No
10	48	27.2	2	Simple Mx + SNB	0	Small seroma	No
11	60	21	2	Simple Mx + ANC	0	Nil	No
12	51	19.7	2	NS Mx + SNB + prepectoral expander reconstruction	1	Nil	No
13	52	25	2	SS Mx + SNB + recon with expander prepec	1	Nil	No
14	53	31.6	2	Simple Mx + ANC	0	Large seroma aspirated	No
15	67	22.3	2	Simple Mx + SNB	0	Mild seroma	No
16	34	28.7	2	Simple Mx + SNB	0	Minor wound breakdown, no RTT	No
17	78	27	2	Simple Mx + ANC	1	Small seroma	No
18	49	21.4	2	NS Mx + SNB + implant recon DTI prepec	1	Small scab on the nipple, no RTT	No
19	84	28.6	3	Simple Mx + SNB	0	Small seroma	No
20	54	27.7	2	NS Mx + SNB + implant recon prepec DTI	1	Nil	No
21	52	25.1	2	SS Mx + SNB + implant recon prepec DTI	0	Nil	No
22	74	26.5	2	Simple Mx + ANC	1	Mild bruising, no RTT	No
23	57	24.4	2	Simple Mx + SNB	0	Nil	No
24	74	31.2	2	Simple Mx + SNB	0	Nil	No
25	74	23	2	SS Mx+ SNB + implant recon prepec DTI	1	Minor healing problem, treated conservatively	No
26	44	23.6	2	NS Mx + SNB + implant (Becker) recon prepec	0	Nil	No
27	67	24.6	2	Simple Mx + SNB	1	Minor wound scab	No
28	82	24.6	2	Simple Mx + SNB	0	Nil	No
29	62	21.5	2	NS Mx + SNB + implant recon prepec DTI	1	Minor wound scab	No
30	83	31	3	Simple Mx	0	Small seroma	No
31	68	23.3	2	Mx + SNB + implant recon prepec DTI	1	Small seroma	No
32	43	17	2	NS Mx + SNB + implant recon prepec DTI	0	Mild nipple ischaemia	No
Mean	60.2	25.8					

Mean NRS for pain at rest and on movement were 1.88 vs. 2.56, 2.03 vs. 2.84, and 1.85 vs. 2.3 out of 10 on POD one, two, and three, respectively, indicating mild pain throughout. Six patients required opioids on POD one, four patients on POD two, and none on the third day. The opioids used were codeine and liquid morphine.

Sleep disturbance was defined as waking up due to pain and was observed in three patients on POD one, five patients on POD two, and three patients on POD three. A total of 11 patients experienced sleep disturbance over the first three days. Seven were awakened once and four were awakened twice due to pain.

The mean satisfaction score with the catheter was 9.25 (SD: 1.18). Of patients, 100% would recommend the technique to other patients.

The QoR-15 data were scored out of a maximum of 150. Of all the patients who were given the QoR-15 questionnaire, only 15 patients returned both the preoperative and postoperative forms. The QoR-15 scores reported as median (quartiles) were 143 (136, 147) preoperatively and 135 (126.5, 143) postoperatively, with a median difference of -3 (95% CI: -6 to 0). Table 2 shows the main pain and recovery parameters.

**Table 2:** Pain scores and recovery parameters and QoR-15 scores NRS: numerical rating scale; (-): not applicable; POD: postoperative day; SD: standard deviation; CI: confidence interval; QoR: Quality of Recovery. * Pain scale 0-10; ** number of episodes in patients.

Outcomes	Baseline (preoperatively)	POD 1	POD 2	POD 3	Total
NRS rest (mean)*	-	1.88	2.03	1.85	-
NRS move (mean)*	-	2.56	2.84	2.3	-
Opioid use (n)	1 (pre-existing)	6/32	4/32	0/32	10
Sleep disturbance** (episodes)	-	4 in 3 patients	7 in 5 patients	4 in 3 patients	15 episodes in 11 patients
Satisfaction with the catheter (mean)	-	-	-	-	9.25/10 (SD: 1.18)
QoR-15 score, median (quartiles)	143 (136,147)	-	135 (126.5, 143)	-	Median difference: -3 (95% CI: -6 to 0)

Regarding the surgical technique, 19 patients had a simple mastectomy and 13 were skin-sparing with or without nipple-sparing mastectomy and immediate implant reconstruction. All reconstructions were in the prepectoral plane. Axillary surgery distribution was as follows: 26 had sentinel node biopsy (SNB), five had axillary node clearance (ANC), and one had no axillary procedure.

The day case rate for mastectomy was 21/32 (66%) and for immediate implant reconstruction was 5/13 (39%). This shows improvement from the previous 6% rate for simple mastectomy and 0% for MIBR in our institution. Only one patient stayed two nights, the rest were either day case or one night stay. There were no readmissions in the first postoperative week.

There were no major complications related to catheter insertion. Minor issues with catheters occurred in four patients. Two patients had leakage from the catheter and required re-dressing by the district nurses. One patient’s catheter dislodged on POD one and one patient attended the emergency department for catheter removal as the district nurse was unable to remove it.

## Discussion

Moderate to severe postoperative pain after mastectomy is a common occurrence and in this quality improvement initiative, we present a novel approach to extend the benefits of regional anaesthesia for these patients. To the best of our knowledge, there are no studies exploring the role of ASPIP in the setting of mastectomy with or without immediate breast reconstruction or the effect of serratus block on length of stay after mastectomy. Our findings demonstrate that the ASPIP provide effective analgesia, enhances the quality of recovery after surgery, and is safe to use in the day case setting as part of our enhanced recovery programme.

SAPB targets the lateral cutaneous branches of the intercostal nerves, providing analgesia to the anterolateral chest wall [14]. This is long known to be effective in breast and thoracic surgery. In this project, we evaluated its efficacy as an extended analgesic technique beyond the first few hours provided by a single shot block. Inserting the catheter intraoperatively is straightforward and adds only a few minutes to the surgical time.

In a randomised clinical study of ultrasound-guided SAPB versus no block in mastectomy patients, Rahimzadeh et al. found significantly lower fentanyl consumption in the SAPB group [22]. In our cohort, patients received continuous SAPB delivered via a catheter and an ambulatory infusion pump, which extended the duration of analgesia and only a few patients required the use of rescue codeine or liquid morphine.

In a retrospective cohort analysis, Chaudhry et al. examined the role of the elastomeric pump on pain control and length of stay following subpectoral immediate expander implant breast reconstruction. The catheter was inserted along the inframammary fold (IMF), not directed at specific nerves. They found significantly shorter length of stay and lower visual analogue scale scores in the group who had the pump compared to the group with standard postoperative analgesia [23]. Our patient cohort experienced only mild pain on NRS at rest and on movement with the use of the ambulatory pump.

In a small retrospective cohort study performed in the UK, there was no report of severe postoperative pain among patients who received serratus plane block intraoperatively [11]. In 2011, the National Mastectomy and Breast Reconstruction Audit showed that 6.2% of the patients were reported to have severe pain after mastectomy [24]. This represents a large number of patients given the number of mastectomies performed.

Bell et al. evaluated the role of Pecs 2 (pectoserratus block) block on day-case mastectomy rates. In their cohort, 82% of patients who had the block were discharged as day cases compared to 10% in the no-block group [25]. In contrast to our cohort, their study was limited to a small number of patients who had simple mastectomy without reconstruction. We have found that immediate implant reconstruction does not add more surgical insult and in fact, the wounds are smaller and better tolerated. Traditionally, the main reason for admitting the patient overnight to the hospital was for the early detection of immediate postoperative surgical complications and not for postoperative pain management.

We found few studies examining day-case or outpatient management in the setting of MIBR. The studies have all established the safety of day surgery immediate breast reconstruction (IBR) with equivalent complication rates to inpatient management while providing improved patient satisfaction. In a large retrospective database review by Qin et al., patients were divided into whether they were managed as inpatient or outpatient (day-case) following mastectomy and immediate expander insertion. The groups were propensity score matched based on preoperative characteristics. Overall, 30-day complication rates and return to theatre (RTT) were similar between the two groups [26]. Similarly, in a small retrospective cohort study, Simpson et al. reported low complication rates of outpatient MIBR [27]. Neither of these papers reported on the use of regional analgesic blocks or the use of postoperative infusion pumps as in our project. In our cohort, none of our patients had any early complication that would have been prevented by an overnight stay in the hospital and there were no unplanned readmissions in the first month.

Dumestre et al. reported a 100% day surgery rate of IBR in 29 patients using an enhanced recovery protocol incorporating standard perioperative education and multimodal analgesia. Local anaesthetic (0.25% bupivacaine with adrenaline) was infiltrated circumferentially around the breast in the subcutaneous plane. This group reported less pain postoperatively and a better quality of recovery [28]. In the UK, Shaker et al. reported an 89% day-case rate in 47 patients who underwent mastectomy and pre-pectoral IBR over a two-year period. This was achieved using a combination of patient education, a well-established day surgery unit, and breast care nurse follow-up. Local anaesthetic blocks were limited to surgeon-administered intercostal nerve blocks intraoperatively [29]. According to the latest UK GIRFT report in 2021 [18], just under 20% of mastectomies in England with no immediate reconstruction were day cases. In this service evaluation, we demonstrated significantly higher day case rates with the use of multimodal analgesia and the use of APSIP. Increasing patient and surgeon awareness is an important factor in increasing day-case rates.

Our project has some limitations. It is a service evaluation and as such the results cannot be generalised. However, it can reaffirm the efficacy of prolonged regional block in managing acute nociceptive pain after mastectomy and improve patient experience and satisfaction. Another limitation is limited participants to provide a strong argument for day-case mastectomy and in particular MIBR. Nevertheless, it can certainly help clinicians make better decisions regarding the safety of day-case mastectomy, as the practice has now become the norm in our unit rather than the exception.

## Conclusions

ASPIP with a continuous infusion of local anaesthetic provided effective postoperative analgesia as part of our enhanced recovery programme for mastectomies. This can be managed safely in the outpatient setting with no significant complications and high patient satisfaction. Our data only cover the immediate postoperative outcomes and further studies are warranted to look at the long-term benefits of ASPIP, including its potential role in the incidence of chronic post-mastectomy pain. We encourage other institutions to consider this technique for improving the day case rates and the quality of recovery after major oncological breast surgery.

## Figures and Tables

**Figure 4:**
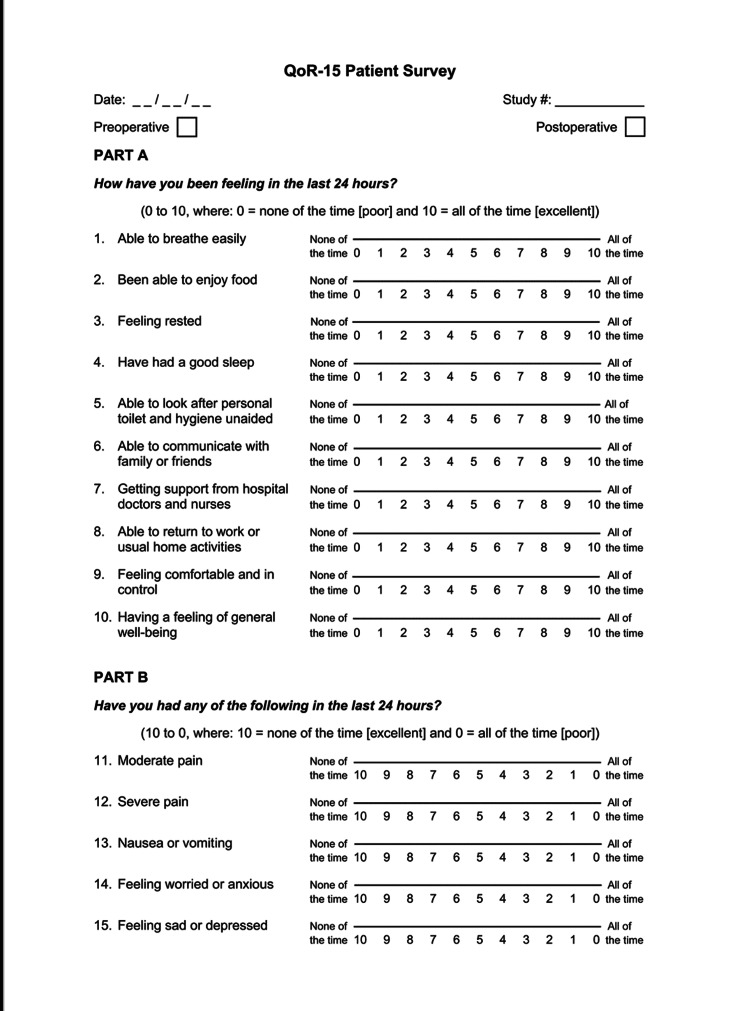
QoR-15 questionnaire QoR-15: Quality of Recovery-15. Courtesy of Stark et al. (2013) [20].

## Appendices

Appendix 1
